# Writers’ uncertainty in scientific and popular biomedical articles. A comparative analysis of the British Medical Journal and Discover Magazine

**DOI:** 10.1371/journal.pone.0221933

**Published:** 2019-09-05

**Authors:** Ramona Bongelli, Ilaria Riccioni, Roberto Burro, Andrzej Zuczkowski

**Affiliations:** 1 Department of Political Science, Communication and International Relations, University of Macerata, Macerata, Italy; 2 Department of Education, Cultural Heritage and Tourism, University of Macerata, Macerata, Italy; 3 Department of Human Sciences, University of Verona, Verona, Italy; Johannes Gutenberg Universitat Mainz, GERMANY

## Abstract

Distinguishing *certain* and *uncertain information* is of crucial importance both in the scientific field in the strict sense and in the popular scientific domain. In this paper, by adopting an *epistemic stance* perspective on certainty and uncertainty, and a mixed procedure of analysis, which combines a bottom-up and a top-down approach, we perform a comparative study (both qualitative and quantitative) of the uncertainty linguistic markers (verbs, non-verbs, modal verbs, conditional clauses, uncertain questions, epistemic future) and their scope in three different corpora: a historical corpus of 80 biomedical articles from the British Medical Journal (BMJ) 1840–2007; a corpus of 12 biomedical articles from BMJ 2013, and a contemporary corpus of 12 scientific popular articles from Discover 2013. The variables under observation are time, structure (IMRaD vs no-IMRaD) and genre (scientific vs popular articles). We apply the Generalized Linear Models analysis in order to test whether there are statistically significant differences (1) in the amount of uncertainty among the different corpora, and (2) in the categories of uncertainty markers used by writers. The results of our analysis reveal that (1) in all corpora, the percentages of uncertainty are always much lower than that of certainty; (2) uncertainty progressively diminishes over time in biomedical articles (in conjunction with their structural changes–IMRaD–and to the increase of the BMJ Impact Factor); and (3) uncertainty is slightly higher in scientific popular articles (Discover 2013) as compared to the contemporary corpus of scientific articles (BMJ 2013). Nevertheless, in all corpora, modal verbs are the most used uncertainty markers. These results suggest that not only do scientific writers prefer to communicate their uncertainty with markers of possibility rather than those of subjectivity but also that science journalists prefer using a third-person subject followed by modal verbs rather than a first-person subject followed by mental verbs such as *think* or *believe*.

## Introduction

Distinguishing certain and uncertain information (i.e. factual vs. speculative, hedged, mitigated information) is of crucial importance both in the scientific field in the strict sense and in the popular scientific domain. In the first case, the communication of a piece of information in a certain or uncertain manner determines opposite outcomes (health policies, clinical practice, etc.). On the other hand, the popular scientific communication (magazines, TV, web, etc.) plays a significant role in spreading scientific knowledge, in making people aware, and in assuming subsequent attitudes and behaviours.

Given the importance of this topic in determining practical decision-making, the study of hedging, uncertainty, mitigation and the like in *scientific writing* has received increasing attention from scholars since the 1990s [1–9]. Recently as well, also researchers in the Natural Language Processing community have focused their attention on the detection of certainty and uncertainty markers and their linguistic scope (e.g. [10–18]). However, these studies tend.

1. to be small in their number of full-text scientific articles (for example, Bioscope [10], one of the premiere corpora annotated for uncertainty, comprises of only by nine full-text articles);
2. to lack a historical perspective to evaluate how uncertainty has evolved over time;
3. to use a top-down analysis procedure for detecting certainty and uncertainty markers (i.e., a predetermined list of such markers taken from grammars, dictionaries, previous relevant studies, etc.);
4. to lack a linguistic theory concerning the communication of certainty and uncertainty.

As for the study of *popular scientific texts*, it has often produced controversial results concerning the use of hedging [19–20]. As Varttala [21–22] states, hedging was prevalently investigated in specialist-to-specialist research articles. His study was the first that compared scientific and popular medicine articles for a number of selected lexical hedging devices. He found that hedges often occur in professional medical articles, but are also typical of popular scientific articles dealing with similar topics.

Choi et al. [23] compared scientific and popular articles regarding the GMO debate and suggesting that hedges occur less frequently in scientific discourse than in popular text. Their findings are consistent with those of Schmied [24], who compared scientific and popular medicine articles, observing that more than double the number of lexical hedges occourred in the latter. These results do not support previous findings [25–29] according to which hedges are more abundant in research articles rather than in their popularized versions.

## Aims

In a previous study [30], we aimed to fill the four above-mentioned gaps in existing the literature. By adopting an *epistemic stance* perspective on certainty and uncertainty (see section Theoretical Framework), and a mixed procedure of analysis, which combines a bottom-up and a top-down approach (see Introduction, and Method in [30]), we identified the uncertainty markers and their linguistic scope (i.e., the rate of uncertainty) in 80 scientific articles from the British Medical Journal (available at PubMed Central, http://www.ncbi.nlm.nih.gov/pmc/journals/3/, last access February 2012), randomly selected from 1840 to 2007 (henceforth, this corpus will be abbreviated as BMJ 1840–2007), in order to test whether the rate of certainty and uncertainty was changed or has remained stable over time. On the basis of the results of this previous study (see next sections), in the present one, we undertake the following:

1. Within BMJ 1840–2007, we compare 22 articles with an IMRaD structure (Introduction, Method, Results, and Discussion) [31–33] with 58 articles with a no-IMRaD structure, in order to verify whether the IMRaD variable affects the percentage of certainty and uncertainty (see section Study 1). Here, the variable under analysis is structural.
2. We identify uncertainty markers and their linguistic scope (i.e., the percentage of uncertainty) in a corpus of 12 scientific articles from the British Medical Journal 2013 (henceforth abbreviated as BMJ 2013), all with an IMRaD structure (see section Study 2). Then, we compare the results with those from the 22 articles having an IMRaD structure in BMJ 1840–2007 in order to verify whether the rate of certainty and uncertainty in articles having an IMRaD structure changed or has remained stable over time (see section Study 3). Here, the variables under analysis are temporal and structural.
3. We identify uncertainty markers and their linguistic scope (i.e., the percentage of uncertainty) in a corpus of 12 popular scientific articles from Discover Magazine 2013 (henceforth abbreviated as Discover 2013) (see section Study 4). Then, we compare the results with those from BMJ 2013 in order to verify if the different genre of the articles (scientific vs. popular) affect the rate of certainty and uncertainty (see section Study 5). Here, the variable under analysis is genre.

## Theoretical framework

The study of certainty and uncertainty in communication is related to more general topics concerning *epistemicity* (e.g., [34–39]), *evidentiality* (e.g., [35; 40–43]), *mitigation* (e.g., [44–45]), *hedging* (e.g., [46–48]), and more specifically *epistemic stance* (e.g., [49–55]).

As described in details previously [30], we adopted the epistemic stance perspective on certainty and uncertainty since it focuses on the speakers/writers in the here and now of communication, i.e., on how they position themselves in terms of certainty and uncertainty with regard to the information they are conveying here and now, while communicating [56].

From this perspective, a piece of information is communicated as certain when, in the here and now of communication, the speaker/writer’s commitment to its truth is at the maximum or a high level:

(1)
  There is a relationship between smoking and lung cancer
(2)
  *There is* ***certainly*** *a relationship between smoking and lung cancer*

Declarative sentences in the indicative mood without (example 1) or with (example 2) a certainty marker, like the adverb *certainly*, are the most used to communicate certainty. In both examples, the authors communicate that they are certain that the piece of information p they are conveying is true, i.e., they are saying that they evaluate p as being true.

Vice-versa, a piece of information is communicated as uncertain when, in the here and now of communication, the speaker/writer’s commitment to its truth is at the minimum or a low level:

(3)
  *There* ***can*** *be a relationship between smoking and lung cancer*
(4)
  *The results of our study*, *tell us that* ***perhaps*** *there is a relationship between smoking and lung cancer*

In examples 3 and 4, verbs like *can*, and adverbs like *perhaps* convey the writers’ uncertain stance towards the information p. In the here and now of communication, they say that they do not know whether p is true or false; therefore, they communicate p as uncertain, i.e., they tell the readers that they are not certain about the truth of p.

In written texts such as BMJ articles, uncertainty markers (from here on UMs) can refer either to the author’s uncertainty or to somebody else’s uncertainty. Both types of uncertainty can refer to the present, past, or future.

As stated above, an essential point in our study on BMJ is the adoption of an epistemic stance perspective: we specifically aimed at identifying the UMs referring to the writer (= the author of the article) in the here and now of his/her communication, i.e., at the time the article was being written.

We excluded from our analysis both the UMs referring to the writer in the past or future

(5)
  It seemed to me that there was a relationship between smoking and lung cancer

and the UMs referring to somebody else apart from the author of the article

(6)
  Doctor Adler supposes that there is a relationship between smoking and lung cancer

In example (5), in the here and now of communication the author remembers, i.e., knows, that there and then (i.e., in the past) he was uncertain about the relationship between smoking and lung cancer. In other words, in the here and now, the author is communicating as certain a piece of information concerning his past uncertainty: it is a certainty communication of a past uncertainty.

In example (6) the author, in the here and now of communication, is communicating as certain a piece of information referring to the uncertainty of someone different from himself.

As a consequence, our analysis only detected “uncertainty under the first case (the author’s uncertainty in the present) and not the other two (the author’s uncertainty in the past or future and somebody else’s uncertainty in the present, past, or future)” (see Linguistic Background in [30]).

To the best of our knowledge, no previous study has applied such distinction in the detection of UMs in the biomedical field. Applying this distinction or not means to study two different types of issues and leads to different quantitative results. When adopting our differentiated approach, only examples 3 and 4 would be considered as uncertain. On the contrary, when adopting an undifferentiated approach, examples 5 and 6 would also be considered as uncertain. The former approach is specific, the latter generic, i.e., it considers any UM indiscriminately. The choice of one or the other approach differently affects the quantitative results concerning both the UMs and their linguist scope, i.e. “the semantic ‘influence’ which such words have on neighbouring parts of a sentence” ([57]: 85). Indeed, in the latter case, the quantitative results would be wider, since the undifferentiated approach considers not only the author’s uncertainty in the present but also in the past and future, as well as the present, past and future uncertainty of somebody else mentioned in the article (for instance, Doctor Adler in example 6).

## Uncertainty markers

In the corpus BMJ 1840–2007, we identified seven categories of UMs, both lexical and morphosyntactic: verbs, non-verbs, modal verbs in the simple present, modal verbs in the conditional mood, if, uncertain questions, and epistemic future (see Table 1).

**Table 1:** UMs categories and sub-categories.

Categories of UMS	Sub-categories of UMS	Examples
**Epistemic verbs**		*I believe*, *we think*, *I suppose*, *it seems…*
**Epistemic non-verbs**	Adjectives	*Possible*, *unlikely…*
	Adverbs	*Perhaps*, *probably…*
	Nouns	*Doubt*, *impression…*
	Personal attributions	*According to my view*, *in my opinion…*
**Modal verbs in the simple present**		*Can*, *may*, *must…* epistemically used
**Modal verbs in the conditional mood**		*could*, *would*, *might*, *should…* epistemically used
**If**	If-clauses (= explicit conditional clauses)	All the if-clause types except for the zero (simple present in both protasis and apodosis; the *if* can be paraphrased by a temporal conjunction, e.g. *when*, *every time*, etc.)
	If-less clauses (= implicit conditional clauses)	“Had I regarded the systolic pressures alone, I should have said that the aortic case had the higher blood pressure…” [58]
	As if / as though	“The extracts behaved as if they contained noradrenaline” [59]
	If / whether introducing indirect uncertain questions	“We have scheduled a 2 year follow up to see if this occurs…” [60]
**Uncertain questions**	Polar interrogatives	“Is there a relationship between smoking and any other cause of death?” [61]
**Epistemic future**	Conjectural use of will	No occurrence found

For further examples and details concerning each category of UMs, see [30].

Here, we only highlight the new categories of UMs found using our theoretical framework and mixed approach. In fact, while the categories *verbs*, *non-verbs*, *modal verbs*, *epistemic future* and the sub-category *if-clauses* within the *if*-category are usually present in the standard lists of UMs used by the authors mentioned in the Introduction, the sub-categories *if-less clauses*, *as if/as though*, *if/whether introducing indirect uncertain questions*, and the category *uncertain questions* are new UMs.

As for the *uncertain questions* category, as described in details in [62], all yes/no questions (polar interrogatives, alternative, tag and declarative questions) are considered uncertain in that they convey *a not-knowing-whether* epistemic stance of the questioner. They present, explicitly or implicitly, two (or more) possible alternatives that the questioner is uncertain about [56; 62–63]. For instance, if the direct question in Table 1 *“Is there a relationship between smoking and any other cause of death*?*”* is transformed into its corresponding indirect form (using the introducing verb *to know* [64]), we have *I do not know whether (or not)* there is a relationship between smoking and any other cause of death.

The *uncertain questions* category includes the *direct* uncertain questions found in the corpus (42 polar interrogatives, see Table 2). The *indirect* uncertain questions are included in the *if*-category, specifically in the sub-category *if/whether introducing indirect uncertain questions*.

**Table 2:** Frequencies and percentages of UMs in BMJ 1840–2007 articles.

UMs categories	Frequencies of UMs	Percentages of UMs
Modal verbs in the simple present	885	31.52
Modal verbs in the conditional mood	714	25.43
Non-verbs	492	17.52
Verbs	362	12.89
If	313	11.14
Uncertain questions	42	1.49
Epistemic future	0	0
**Total**	**2808**	**100**

*Frequencies* indicate the total number of occurrences of UMs (referring to the corresponding category) in the whole corpus (80 papers). Percentages have been calculated over such frequencies.

The sub-category *if-less clauses* includes the conditional constructions having instead of the explicit *if*, only the subject-verb inversion in the protasis. In the example in Table 1, the initial expression with the subject-verb inversion *“Had I regarded…”* is equivalent to *If I had regarded…*

The sub-category *as if/as though* includes comparative constructions introduced by *as if/as though*. In a statement of the form *p as if q*, a comparison between the main clause p and an if-clause q with understood apodosis is established [65–67]: *The extracts behaved as if they contained noradrenaline* = *The extracts behaved as (they would) if they contained noradrenaline* = *If the extracts contained noradrenaline*, *they would behave as they did*.

In conclusion, as shown by the examples in Table 1, our notion of uncertainty includes, in addition to the narrow sense of *uncertainty* (*I do not know whether p; I’m not certain that p; I’m uncertain about p; etc*.), also *possibility* (as expressed, for example, by the epistemic use of the modal verbs and expressions such as *it is possible/probable*, etc.) and *subjectivity* (i.e., the communication of the writers’ point of views, such as the expressions *in my opinion*, *according to my view*, *I think*, etc.). “Since these three concepts partially overlap, we prefer using the more generic term “uncertainty”, which encompasses them all” ([34]: 58).

As shown in Table 2, modal verbs in the simple present and in the conditional mood are the most used UMs; non-verbs are more numerous than verbs.

### Scope of uncertainty markers and percentage of uncertainty

The linguistic scope of a UM extends either over a whole sentence (whether including coordinate and subordinate clauses or not) or over a part of it. For instance, example 3, in the section Theoretical Framework, was entirely tagged as uncertain (= 10 words). In example 4, instead, only the subordinate clause (*“…perhaps there is a relationship between smoking and lung cancer”*) was tagged as uncertain (= 10 words). The preceding clause (*“The results of our study tell us that”*) was tagged as certain (= 8 words). Following the same criterion, examples 1 and 2 were also tagged as certain (= 9 and 10 words respectively). This means that, in principle, what was not tagged as uncertain was tagged as certain, since the notions of epistemicity and epistemic stance include only two dimensions: certainty and uncertainty.

As shown in Table 3, the percentage of uncertainty (UMs + their scope) is always much lower than that of certainty both in each period and in the whole corpus. Specifically, in the whole corpus, the uncertainty is 20% and certainty is 80%.

**Table 3:** Certainty and uncertainty tokens and percentages for each period.

	Tokens			Percentages		
Periods	Certainty	Uncertainty	Total	Certainty	Uncertainty	Total
1^st^ Period	39,647	7,446	47,093	84.18	15.81	100
2^nd^ Period	40,897	11,018	51,915	78.77	21.22	100
3^rd^ Period	41,973	12,545	54,518	76.98	23.01	100
4^th^ Period	28,685	5,671	34,356	83.49	16.50	100
**Total**	**151,202**	**36,680**	**187,882**	**80.47**	**19.52**	**100**

### Statistical analysis

As described in details previously [30], in order to test if there were significant differences in the amount of certainty and uncertainty tokens along the four periods, the Generalized Linear Models (GLM) [68] and Wald *χ*2 tests on GLM [69] were applied.

As shown in Table 3, the percentage of uncertainty in the four periods ranges from 16 to 23% in a non-significant way. The analysis did not reveal any significant variation, even with regards to the amount of certainty.

This means that the percentage of certainty (80%) and uncertainty (20%) is the same over the 167-year span. Scientific writers have been using uncertainty in an unaltered manner and always in a smaller percentage as compared to certainty.

## The new five studies

As stated in the section Aims, in the following five studies, we aimed to ascertain if there were significant variations in the percentages of certainty and uncertainty along *time*, between different *structures* of the articles (IMRaD vs no-IMRaD), and between different *genres* (scientific vs popular). In other words, we take into consideration only three main variables: time, structure, and genre.

Other possible variables, such as the specific topic of each article (cancer, small-pox, etc.) and the methods used by the writers (experimental, meta-analysis, etc.) fall beyond the aims of the present paper.

## Study 1. Comparative analysis of IMRaD and no-IMRaD articles in BMJ 1840–2007

### Corpus, aims, procedures

The statistical analysis of the temporal variable in BMJ 1840–2007 (see section Statistical Analysis) revealed no significant differences in the percentage of certainty and uncertainty over time. However, the BMJ 1840–2007 corpus consists of scientific articles with different structures. Of the 80 articles, 22 have an IMRaD structure, while 58 do not. Out of the 22 IMRaD articles, 11 have been identified in the third period (1921–1960) and 11 in the fourth (1961–2007). In Study 1, we compare the sub-corpus of 22 IMRaD articles with the sub-corpus of 58 no-IMRaD articles in order to verify if this structural variable can determine significant differences in the rate of uncertainty.

### Results

#### Uncertainty markers

The most used UMs (modal verbs in the simple present and in the conditional mood) are the same in the two sub-corpora as well as in the whole corpus, independently from the structural variable (IMRaD vs. no-IMRaD). Non-verbs are more numerous than verbs (see Table 4).

**Table 4:** Frequencies and percentages of UMs in the BMJ 1840–2007 corpus, and in IMRaD and no-IMRad articles.

UMs categories	1840–2007		No-IMRaD articles		IMRaD articles	
	Frequencies	Percentages	Frequencies	Percentages	Frequencies	Percentages
Modal verbs in the simple present	885	31.52	648	33.01	237	28.04
Modal verbs in the conditional mood	714	25.43	486	24.75	228	26.98
Non-verbs	492	17.52	310	15.79	182	21.53
Verbs	362	12.89	260	13.24	102	12.07
If	313	11.14	224	11.41	89	10.53
Uncertain questions	42	1.49	35	1.78	7	0.82
**Total**	**2808**	**100**	**1963**	**100**	**845**	**100**

#### Scope

The percentage of certainty and uncertainty in the 22 IMRaD articles is 82% and 18% respectively, while in the 58 no-IMRaD articles, it is 80% and 20%, as with the whole corpus.

This means that the uncertainty in IMRaD articles is of 2 percentage points lower than that in no-IMRaD articles.

#### Statistical analysis

**Fig 1:**
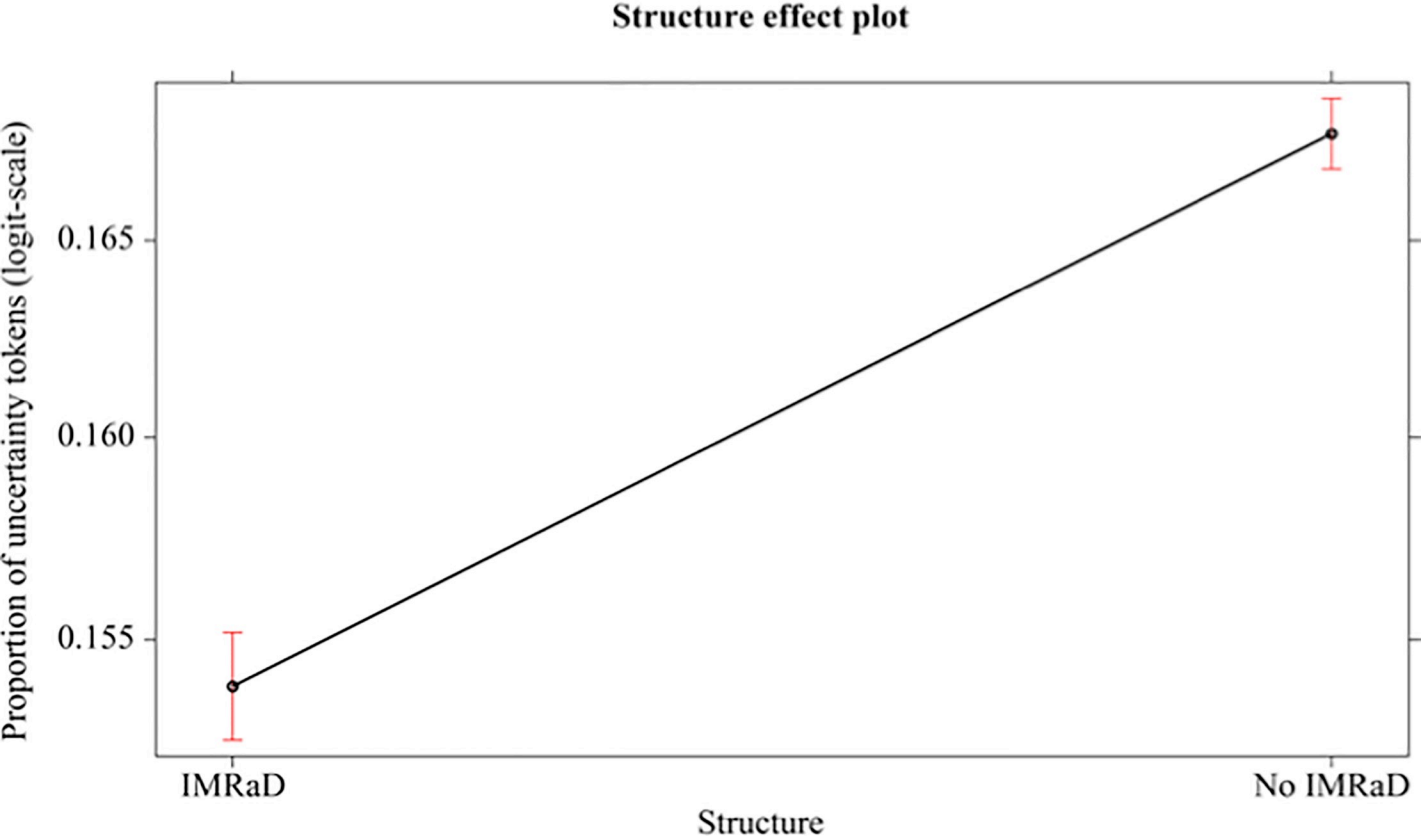
Mean proportions (95% confidence interval) of uncertainty tokens (in logit-scale) of different structure (IMRaD, no-IMRad, BMJ 1840–2007).

In all statistical analyses, the responses were first analysed applying the Generalized Linear Models (GLM), using proportion of uncertainty tokens as the dependent variable. Subsequently, GLM was applied using proportion of UMs as the dependent variable. For this reason, we used GLM with the logit link function and binomial family. Precisely, we perform ANOVA Tables (Type 3) via Wald *χ*2 tests implemented in the R-software “car” package [69]. Bonferroni corrections were applied to post-hoc comparisons. In the first following analysis, the independent factor is structure in BMJ 1840–2007: the difference between IMRaD structure vs no-IMRad structure is significative (*χ*^2^_(1, N = 80)_ = 69.421, p < 0.001, Cohen’s d = 0.93). See Fig 1.

**Fig 2:**
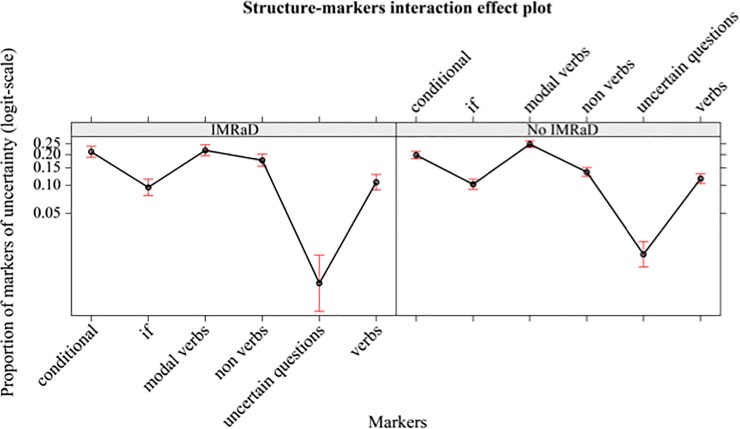
Mean proportions (95% confidence interval) of markers of uncertainty (in logit-scale) of different markers (conditional, if, modal verbs, non-verbs, uncertain questions, verbs), separately by structure (IMRaD, no-IMRaD, BMJ 1840–2007).

There are no significant effects due to the interaction between IMRaD vs no-IMRad structure in BMJ 1840–2007 and UMs. See Fig 2.

This means that in both sub-corpora the writers use the same categories of UMs in similar proportion.

#### Summary of the main results

In IMRaD articles, the uncertainty is 18% and certainty 82%. In no-IMRaD articles, the uncertainty is 20% and certainty 80%. This means that IMRaD articles are less uncertain than no-IMRaD ones and such a difference is statistically significant. The difference concerning UMs in IMRaD and no-IMRaD articles is statistically not significant.

## Study 2. Uncertainty in BMJ 2013

### Corpus

12 research articles (one for each month of 2013, which was the last year available when this study started) with the IMRaD structure were randomly selected from the “full online Archive” (http://www.bmj.com/archive of the British Medical Journal (BMJ), section “Research”). Total tokens: 55,198; average number of tokens per article: 4,599.

### Aims

1. To identify which and how many lexical and morphosyntactic UMs are used by writers in order to communicate their own uncertainty.
2. To identify how much uncertainty (UMs + their scope) is present in each article and in the whole corpus.

### Procedures of analysis

The articles were preliminary manually edited and converted from the .pdf extension source into plain text files (.txt). The corpus included titles and main texts. Reference lists, figures, tables, authors’ names and affiliations, etc. were excluded to facilitate the data set processing.

The qualitative analysis was performed by three independent judges: K coefficient was calculated between two of them and resulted in 0,93 for the UMs identification and 100 for the scope.

### Results

#### Uncertainty markers

Modal verbs in the conditional mood are the most used UMs (Table 5).

**Table 5:** Frequencies and percentages of UMs in BMJ 2013.

UMS categories	Frequencies	Percentages
Modal verbs in the conditional mood	123	34.07
Non-verbs	91	25.21
Modal verbs in the simple present	84	23.27
Verbs	34	9.42
If	28	7.76
Uncertain questions	1	0.28
Epistemic future	0	0
**Total**	**361**	**100**

Among modal verbs in the conditional mood, *could* and *would* are the most used (Table 6).

**Table 6:** Frequencies and percentages of modal verbs in the conditional mood.

Modal verbs in the conditional mood	Frequencies	Percentages
could + could not	43 + 1 = 44	35,77
would + would not	27 + 4 = 31	25,20
should + should not	22 + 3 = 25	20,33
might + might not	20 + 3 = 23	18,70
**Total**	**123**	**100**

Among non-verbs, *likely*, *potentially*, *potential*, *possible* and *probably* are the most used (Table 7).

**Table 7:** Frequencies and percentages of non-verbs.

Non-verbs	Frequencies	Percentages
likely	15	16.48
potentially	14	15.38
potential	9	9.89
possible	7	7.69
probably	7	7.69
possibility	5	5.49
to our knowledge	5	5.49
unclear	5	5.49
perhaps	4	4.40
possibly	4	4.40
apparently	3	3.30
uncertainty	3	3.30
uncertain	2	2.20
unlikely	2	2.20
apparent	1	1.10
assumption	1	1.10
confident	1	1.10
hypothesis	1	1.10
likelihood	1	1.10
plausibly	1	1.10
**Total**	**91**	**100**

Among modal verbs in the simple present, *may* and *can* are the most used (Table 8).

**Table 8:** Frequencies and percentages of modal verbs in the simple present.

Modal verbs in the simple present	Frequencies	Percentages
May	59	70.24
Can	22	26.19
May not	3	3.57
**Total**	**84**	**100**

Among verbs, *suggest/s* and *seem/s* are the most used (Table 9).

**Table 9:** Frequencies and percentages of verbs in the simple present.

Verbs	Frequencies	Percentages
suggest/s	19	55.88
seem/s	7	20.59
suggesting	3	8.82
assuming	2	5.88
we think	2	5.88
expect	1	2.94
**Total**	**34**	**100**

Within the *If category*, *if/whether* is the most used sub-category (Table 10).

**Table 10:** Frequencies and percentages of if.

If	Frequencies	Percentages
if/whether	16	57.14
if clauses	7	25
as if	5	17.86
if less	0	0
**Total**	**28**	**100**

#### Scope

**Fig 3:**
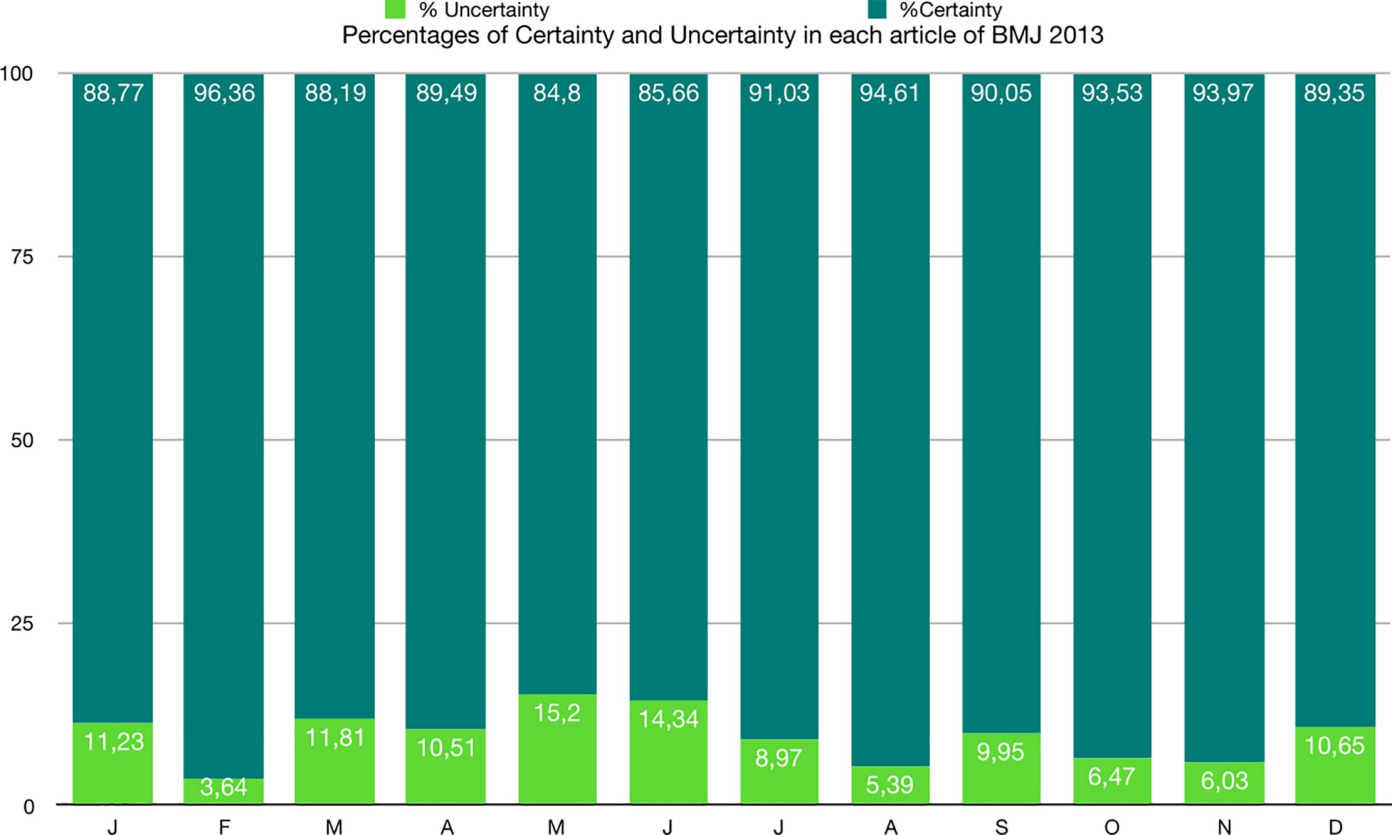
Percentages of certainty and uncertainty in each article of BMJ 2013.

The percentage of uncertainty is much lower than that of certainty, both in each article and in the whole corpus (Fig 3 and Table 11).

**Table 11:** Percentages of certainty and uncertainty in each article and in the whole corpus of BMJ 2013.

	Tokens			Percentages		
Articles	Certainty	Uncertainty	Total	Certainty	Uncertainty	Total
January	3619	458	4077	88.77	11.23	100
February	5080	192	5272	96.36	3.64	100
March	3429	459	3888	88.19	11.81	100
April	3722	437	4159	89.49	10.51	100
May	4001	717	4718	84.80	15.20	100
June	2514	421	2935	85.66	14.34	100
July	3422	337	3759	91.03	8.97	100
August	4560	260	4820	94.61	5.39	100
September	3204	354	3558	90.05	9.95	100
October	4716	326	5042	93.53	6.47	100
November	8157	523	8680	93.97	6.03	100
December	3833	457	4290	89.35	10.65	100
**Total**	**50257**	**4941**	**55198**	**91.05**	**8.95**	**100**

**Fig 4:**
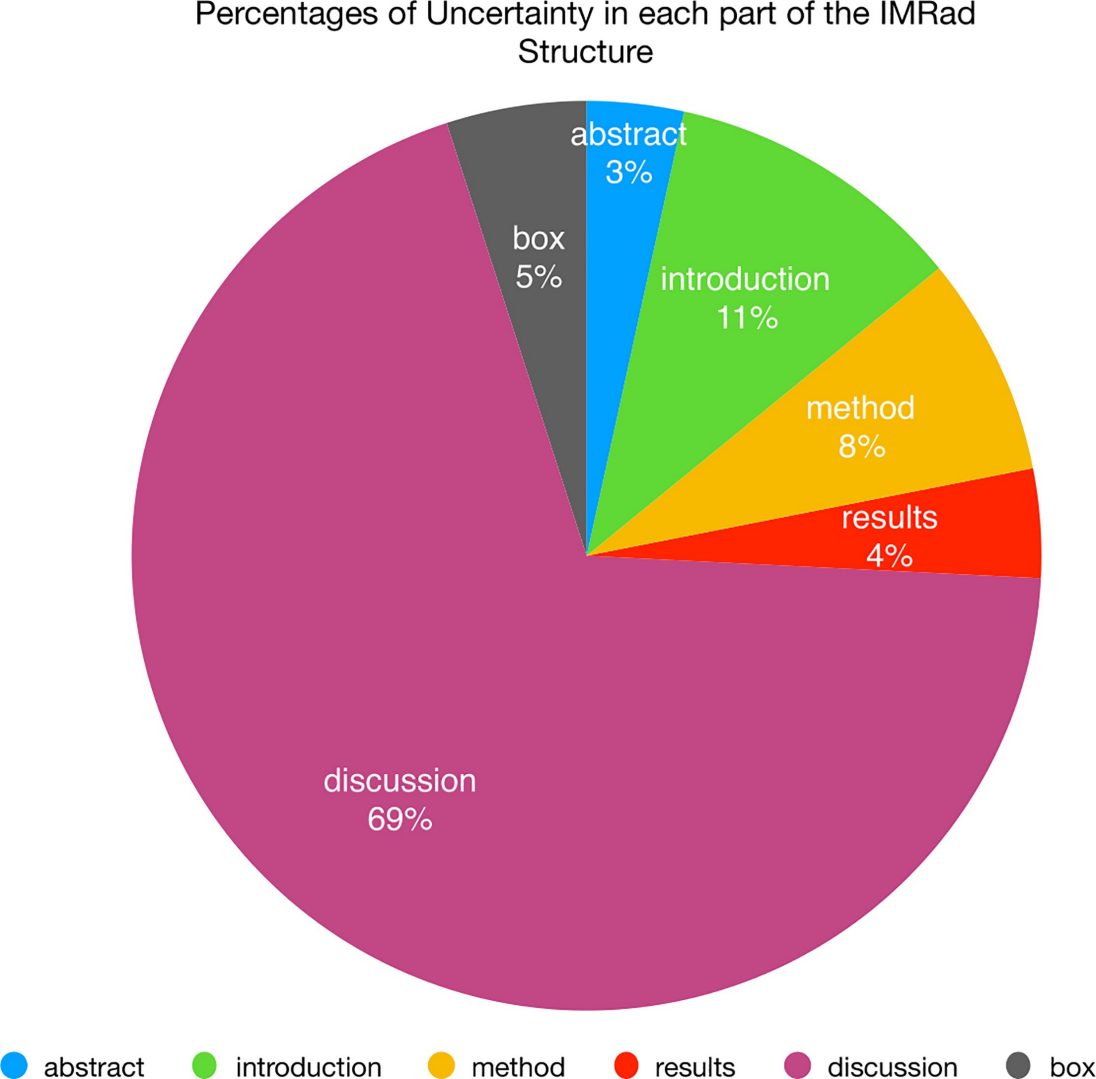
Percentages of uncertainty in each part of IMRaD structure of BMJ 2013 articles.

As shown in Fig 4, of the different sections that form the IMRaD structure (Introduction, Method, Results, and Discussion, to which Box and Abstract have been recently added in BMJ articles), the uncertainty is firstly communicated in the Discussion section (69%), and secondly in the Introduction section (11%).

#### Summary of the main results

The uncertainty communicated in the BMJ 2013 is about 9% of the total, and it is mainly conveyed through modal verbs, both in the conditional (34.07%) and in the simple present (23.26%). If we take them together, they represent more than 50% of all UMs. Non-verbs are used three times more than verbs.

Consistent with the results of many studies on hedges (see for example, [21; 70–71]), the uncertainty is massively present in the Discussion section.

## Study 3. Comparative analysis of IMRaD articles from BMJ 1840–2007 and IMRaD articles from BMJ 2013

### Corpus, aims, procedures

In Study 3, we compare the 22 IMRaD articles from BMJ 1840–2007 with the 12 IMRaD articles from BMJ 2013 in order to determine if there are significant differences in the percentage of uncertainty. In this study, the variable under observation is temporal.

### Results

#### Uncertainty markers

Table 12 shows that the most used UMs are always modal verbs. In the 12 IMRaD articles from BMJ 2013, modal verbs in the conditional mood occur more than modal verbs in the simple present, while in the 22 IMRaD articles from BMJ 1840–2007, the latter were more than the former.

**Table 12:** Frequencies and percentages of UMs in IMRaD BMJ 1840–2007 articles and in IMRaD BMJ 2013 articles.

UMs categories	IMRaD BMJ 1840–2007		IMRaD BMJ 2013	
	Frequencies	Percentages	Frequencies	Percentages
modal verbs in the simple present	237	28.04	84	23.26
modal verbs in the conditional	228	26.98	123	34.07
non-verbs	182	21.53	91	25.20
verbs	102	12.07	34	9.41
if	89	10.53	28	7.75
uncertain questions	7	0.82	1	0.27
**Total**	**845**	**100**	**361**	**100**

#### Scope

The percentage of certainty and uncertainty in the 12 IMRaD articles from BMJ 2013 is 91% and 9% respectively, while in the 22 IMRaD articles from BMJ 1840–2007, it is 82% and 18%. This means that the uncertainty in the former corpus from BMJ 2013 is of 9 percentage points lower than that in the latter sub-corpus from BMJ 1840–2007.

#### Statistical analysis

**Fig 5:**
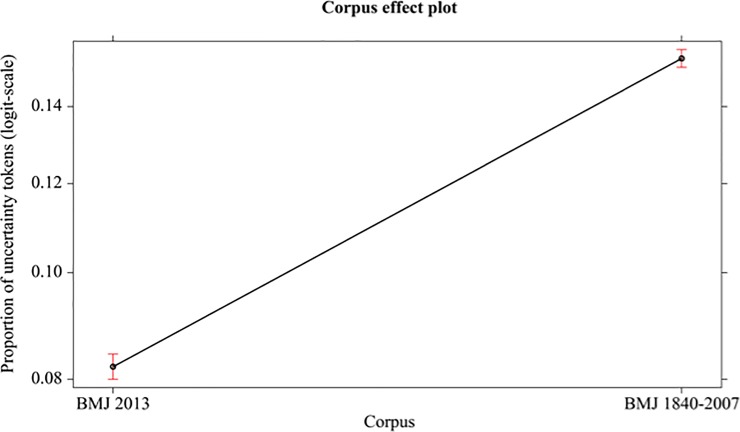
Mean proportions (95% confidence interval) of uncertainty tokens (in logit-scale) of different corpus (BMJ 2013, BMJ 1840–2007 IMRaD articles).

In the following analysis, the independent factor is IMRaD articles: the difference between IMRaD articles from BMJ 1840–2007 and IMRaD articles from BMJ 2013 is significative (*χ*^2^_(1, N = 34)_ = 1624.5, p < 0.001, Cohen’s d = 6.71). See Fig 5.

**Fig 6:**
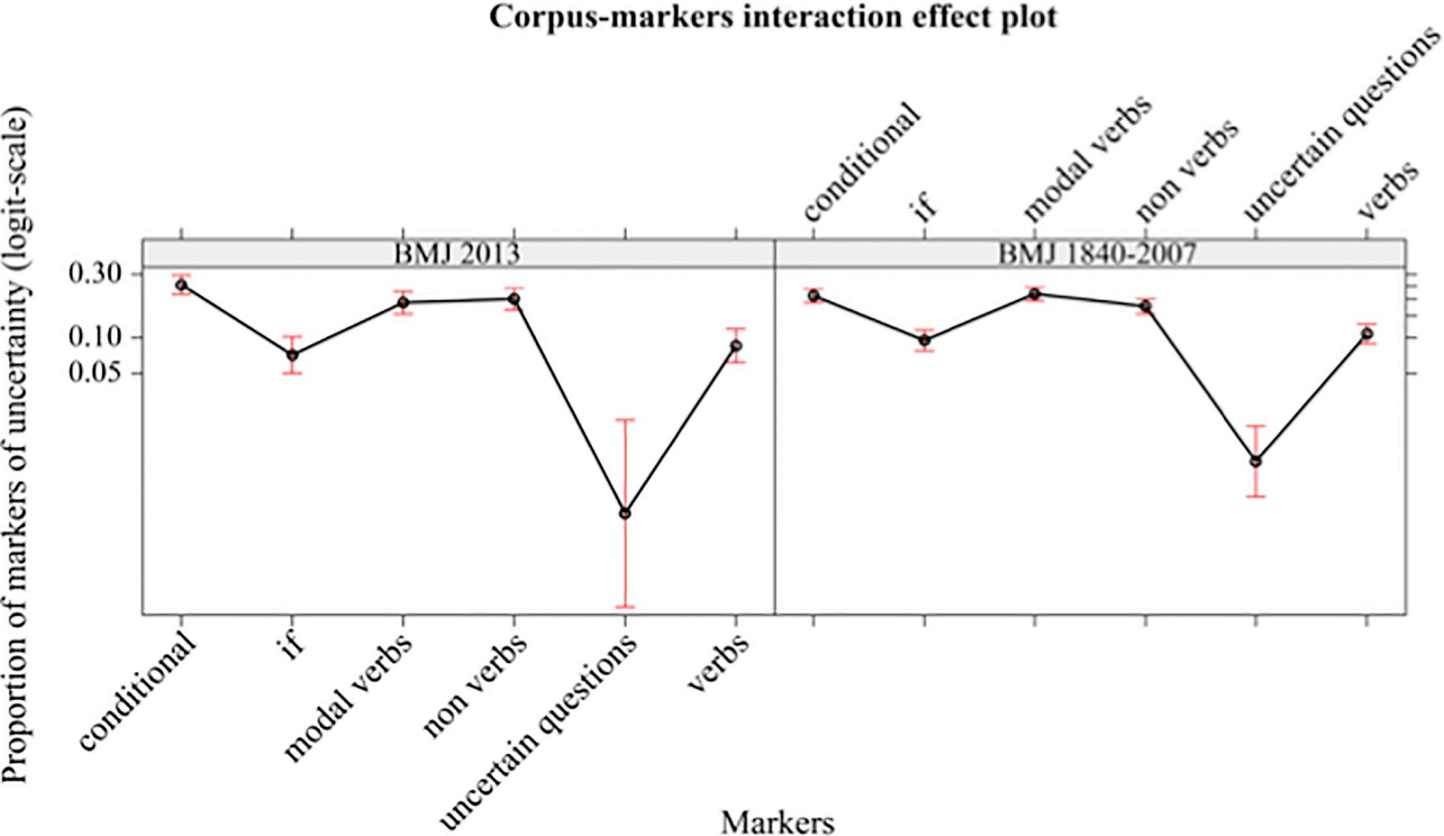
Mean proportions (95% confidence interval) of markers of uncertainty (in logit-scale) of different markers (conditional, if, modal verbs, non-verbs, uncertain questions, verbs), separately by corpus (BMJ 2013, BMJ 1840–2007 IMRaD articles).

There are no significant effects due to the interaction between IMRaD articles from BMJ 1840–2007 and IMRaD articles from BMJ 2013 and UMs. See Fig 6.

This means that in both corpora the writers use the same categories of UMs in similar proportion.

#### Summary of the main results

In IMRaD articles from BMJ 1840–2007, the uncertainty is 18% and certainty 82%. In IMRaD articles from BMJ 2013, uncertainty is 9% and certainty 91%. This means that IMRaD articles from BMJ 2013 are less uncertain than IMRaD articles from BMJ 1840–2007 and this difference is statistically significant. With regard to the UMs, the analysis does not reveal any significant differences.

## Study 4. Uncertainty in Discover 2013

### Corpus, aims, procedures

12 popular articles (one for each month of 2013) were randomly selected from Discover http://discovermagazine.com, section Health & Medicine http://discovermagazine.com/topics/health-medicine.

Total tokens: 36,559; average number of tokens per article 3,046. These popular articles, by definition, have an unconstrained structure, different from the scientific ones (IMRaD).

Aims (identifying UMs + their scope), preliminary procedures and procedures of analysis are the same as those of BMJ 2013. Only the K coefficient was slightly different: 0,89 for the UMs identification and 100 for the scope.

### Results

#### Uncertainty markers

Modal verbs in the conditional mood and in the simple present are the most used UMs (Table 13).

**Table 13:** Frequencies and percentages of UMs in Discover 2013.

UMs categories	Frequencies	Percentages
Modal verbs in the conditional mood	121	33.15
Modal verbs in the simple present	98	26.85
If	46	12.60
Non-verbs	41	11.23
Verbs	33	9.04
Uncertain questions	25	6.85
Epistemic future	1	0.27
**Total**	**365**	**100**

Among modal verbs in the conditional mood, *would* and *could* are the most used (Table 14).

**Table 14:** Frequencies and percentages of modal verbs in the conditional mood.

Modal verbs in the conditional mood	Frequencies	Percentages
would + would not	49+2 = 51	42.15
could	33	27.27
might	32	26.45
should + should not	4+1 = 5	4.13
**Total**	**121**	**100**

Among modal verbs in the simple present, *can* and *may* are the most used (Table 15).

**Table 15:** Frequencies and percentages of modal verbs in the simple present.

Modal verbs in the simple present	Frequencies	Percentages
Can	55	56.12
May	41	41.84
Must	2	2.04
**Total**	**98**	**100**

Within the *if* category, if clauses is the most used sub-category (Table 16).

**Table 16:** Frequencies and percentages of if.

If	Frequencies	Percentages
if clauses	21	45.65
if/whether	21	45.65
as if	3	6.52
if less	1	2.17
**Total**	**46**	**100**

Among non-verbs, *likely*, *probably* and *perhaps* are the most used (Table 17).

**Table 17:** Frequencies and percentages of non-verbs.

Non-verbs	Frequencies	Percentages
likely/more likely	12	29.27
probably	9	21.95
perhaps	6	14.63
maybe	3	7.33
possible	3	7.32
possibility	2	4.88
seemingly	2	4.88
likelihood	1	2.44
not likely	1	2.44
plausibility	1	2.44
possibly	1	2.44
**Total**	**41**	**100**

Among verbs, *seem/s* and *suggest/s* are the most used (Table 18).

**Table 18:** Frequencies and percentages of verbs.

Verbs	Frequencies	Percentages
seem/s	12	36.36
suggest/s	11	33.33
appear	2	6.06
looks	2	6.06
suspect/suspected	2	6.06
do not seem	1	3.03
no one has proven	1	3.03
being not sure	1	3.03
suggesting	1	3.03
**Total**	**33**	**100**

As for uncertain questions, they amount to about 7% of the total UMs, as shown in Table 13.

#### Scope

**Fig 7:**
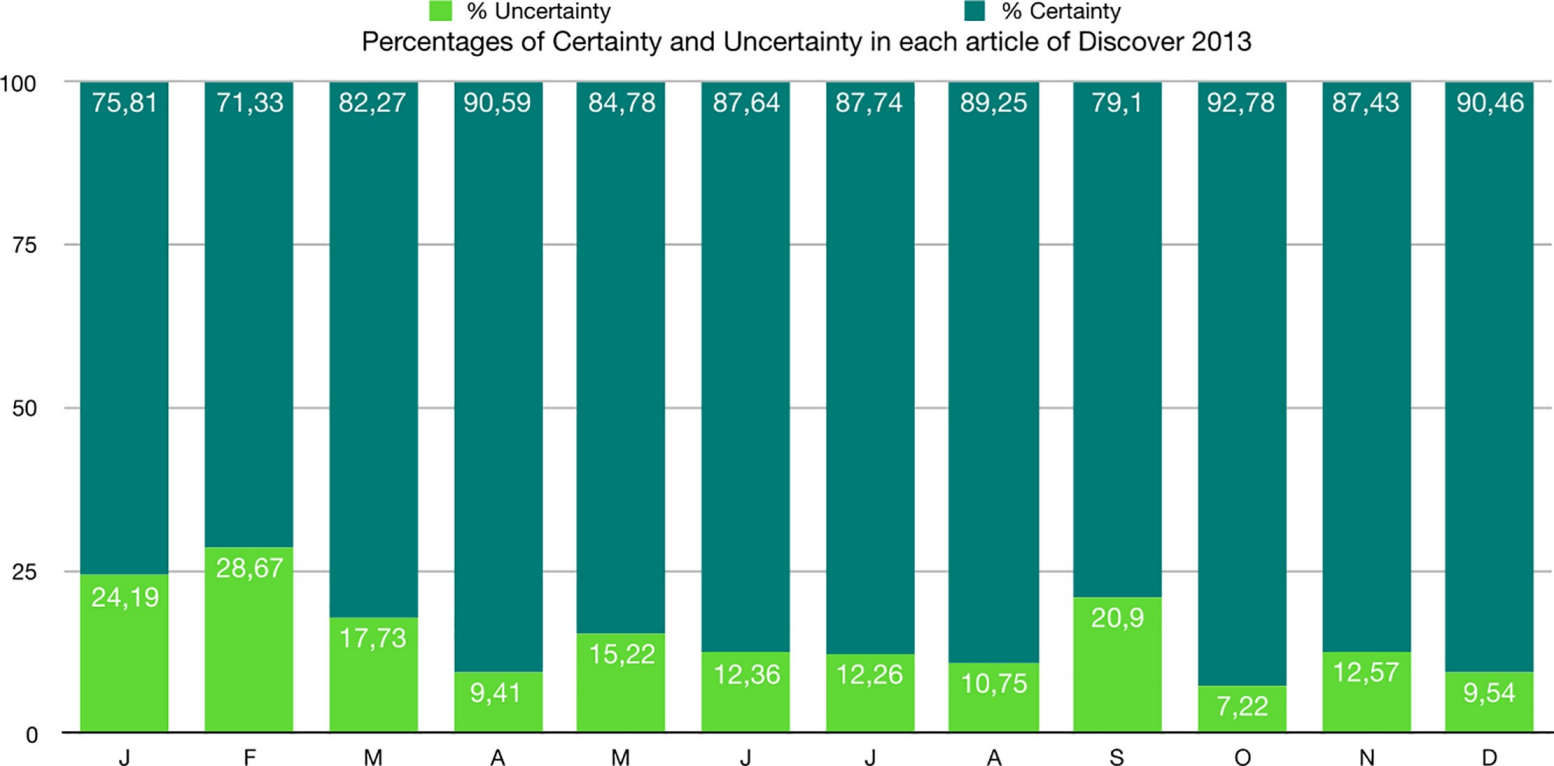
Percentages of certainty and uncertainty in each article of Discover 2013.

The percentages of uncertainty are much lower than that of certainty both in each article and in the whole corpus (Fig 7 and Table 19)

**Table 19:** Percentages of certainty and uncertainty in each article and in the whole corpus of Discover 2013.

	Tokens			Percentages		
Articles	Certainty	Uncertainty	Total	Certainty	Uncertainty	Total
January	351	112	463	75.81	24.19	100
February	316	127	443	71.33	28.67	100
March	1763	380	2143	82.27	17.73	100
April	2880	299	3179	90.59	9.41	100
May	3114	559	3673	84.78	15.22	100
June	3261	460	3721	87.64	12.36	100
July	3229	451	3680	87.74	12.26	100
August	3256	392	3648	89.25	10.75	100
September	984	260	1244	79.10	20.90	100
October	4678	364	5042	92.78	7.22	100
November	4126	593	4719	87.43	12.57	100
December	4165	439	4604	90.46	9.54	100
**Total**	**32123**	**4436**	**36559**	**87.87**	**12.13**	**100**

#### Summary of the main results

The uncertainty communicated in the Discover 2013 is about 12% of the total and is mainly conveyed through modal verbs, both in the conditional (33.15%) and in the simple present (26.84%). If considered together, they represent about 60% of all UMs.

## Study 5. Comparative analysis of Discover 2013 and BMJ 2013

### Corpus, aims, procedures

In Study 5, we compare the 12 BMJ 2013 articles with the 12 Discover articles in order to verify if text genre variable (i.e., scientific vs. popular) could determine significant differences in the percentage of uncertainty. In this study, the variable under observation is genre.

### Results

#### Uncertainty markers

Table 20 shows that the most used UMs are again modal verbs in the conditional mood and in the simple present both in the scientific and popular corpus.

**Table 20:** Frequencies and percentages of UMs in BMJ 2013 and Discover 2013.

UMs categories	BMJ 2013		Discover 2013	
	Frequencies	Percentages	Frequencies	Percentages
Modal verbs in the conditional mood	123	34.07	121	33.15
Modal verbs in the simple present	84	23.26	98	26.84
Non-verbs	91	25.20	41	11.23
Verbs	34	9.41	33	9.04
If	28	7.75	46	12.60
Uncertain questions	1	0.27	25	6.84
Epistemic future	0	0	1	0.27
**Total**	**361**	**100**	**365**	**100**

#### Scope

The percentage of certainty and uncertainty in the 12 articles from BMJ 2013 is 91% and 9% respectively, while in the 12 articles from Discover 2013, it is 88% and 12%. This means that the uncertainty in the former corpus from BMJ 2013 is 3 percentage points lower than that in the latter sub-corpus from Discover 2013.

#### Statistical analysis

**Fig 8:**
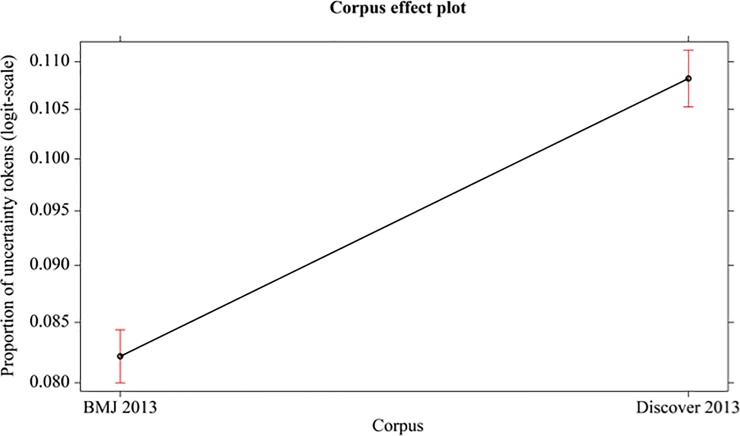
Mean proportions (95% confidence interval) of uncertainty tokens (in logit-scale) of different corpus (BMJ 2013, Discover 2013).

In the following analysis, the independent factor is corpus 2013. The difference between Discover 2013 and BMJ 2013 is significant (*χ*^2^_(1, N = 24)_ = 194.12, p < 0.001, Cohen’s d = 2.85). See Fig 8.

**Fig 9:**
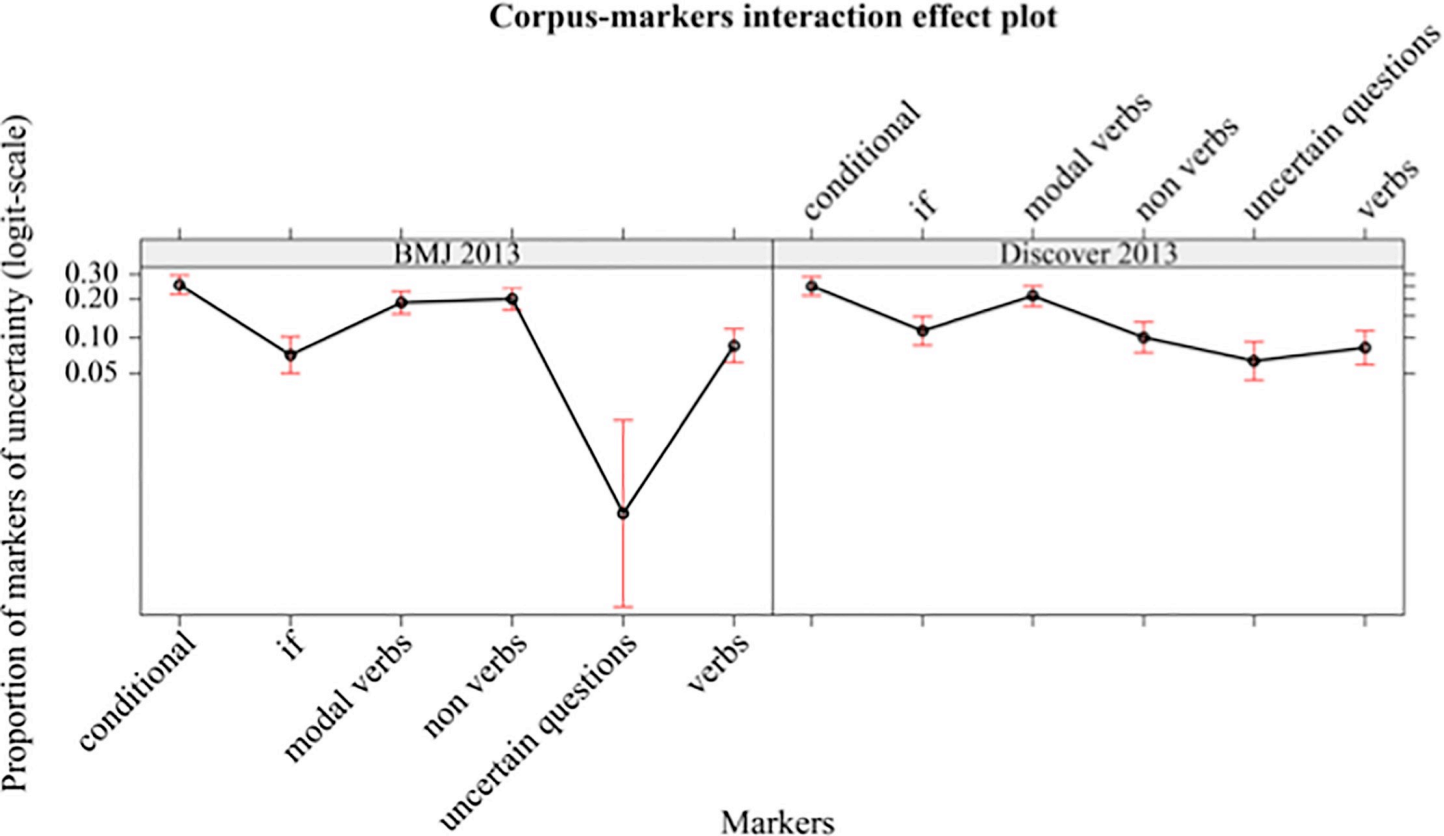
Mean proportions (95% confidence interval) of markers of uncertainty (in logit-scale) of different markers (conditional, if, modal verbs, non-verbs, uncertain questions, verbs), separately by corpus (BMJ 2013, Discover 2013).

There is only one significant effect due to the interaction between corpus discover 2013 –corpus BMJ 2013 and UMs (non-verbs—BMJ 2013 > non-verbs—Discover 2013; z-ratio = 3.997, p = 0.004, Cohen’s d = 0.81). See Fig 9.

#### Summary of the main results

The percentage of certainty and uncertainty in BMJ 2013 is 91% and 9% respectively, while in Discover 2013, it is 88% and 12%. This means that the uncertainty communicated in Discover 2013 is 3 percentage points more than that in BMJ 2013.

In both corpora, the uncertainty is mainly conveyed through modal verbs, both in the conditional and the simple present. If considered together, they represent about 60% of all UMs. Also, the percentage of verbs in both corpora are almost the same (about 9%).

In Discover 2013, the uncertainty is also communicated through uncertain questions, as opposed to in BMJ 2013, which has no occurrence of such UMs.

Non-verbs decrease notably in Discover 2013; the difference between them and the non-verbs in BMJ 2013 is statistically significant.

## Conclusion and discussion

Do time, structure, and genre affect the proportion of certainty and uncertainty in biomedical scientific and popular articles?

Which and how many markers are used in order to communicate uncertainty?

These were the main research questions we tried to answer in the present paper.

The main novelties concern the following:

1. *Theory*: the adoption of the epistemic stance perspective (the UMs detected were only those referring to the writer’s uncertainty in the here and now of writing the article).
2. *Methodology*: the adoption of a mixed procedure for the UMs detection, which combines a bottom-up and a top-down approach.
3. *Results*: the explicit addition of two new categories of UMs, namely the *if* category and the *uncertain questions*.

With regard to the first research question, Table 21 shows that in all corpora (three scientific and one popular), the percentage of certainty is always much higher than that of uncertainty, ranging from 80% (BMJ 1840–2007) to 91% (BMJ 2013). Inversely, the uncertainty ranges from 9% (BMJ 2013) to 20% (BMJ 1840–2007).

**Table 21:** Percentages of certainty and uncertainty in scientific and popular corpora.

	Corpora	% Certainty	% Uncertainty
**Scientific**	**BMJ 1840–2007**	80%	20%
	**BMJ 1840–2007 (IMRaD articles)**	82%	18%
	**BMJ 2013**	91%	9%
**Popular**	**Discover 2013**	88%	12%

As for the second research question, in all corpora, both scientific and popular, the most used UMs are modal verbs, both in the simple present and the conditional mood. These results suggest that not only do scientific writers prefer to communicate their uncertainty with markers of possibility rather than with markers of subjectivity [5; 29] but science journalists also prefer using a third-person subject followed by modal verbs, such as *may* or *could*, rather than using a first-person subject followed by verbs such as *think* or *believe* [72].

In both contexts (scientific and popular), a cautious way (using possibility markers) of communicating a piece of information indeed seems more appropriate than an explicit personal way (using subjectivity markers).

### Scientific corpora

Within the same scientific genre, we took *time* and *structure* as the main two variables under observation. When only the first variable (time) is studied, the percentages of uncertainty (20%) and certainty (80%) remain stable over 167 years, as shown by the results from the corpus BMJ 1840–2007. However, when the second variable (structure) is introduced, the percentages of uncertainty significantly decrease.

In particular, in Study 1, we compared IMRaD and no-IMRaD articles in BMJ 1840–2007. In IMRaD articles, the uncertainty is 18% and certainty 82%. In no-IMRaD articles, the uncertainty is 20% and certainty 80%. This means that IMRaD articles are less uncertain than no-IMRaD ones and this difference is statistically significant.

In Study 3, we compared IMRaD articles from BMJ 1840–2007 and IMRaD articles from BMJ 2013. In the former corpus, the uncertainty is 18% and certainty 82%, while in the latter, uncertainty is 9% and certainty 91%. This means that IMRaD articles from BMJ 2013 are less uncertain than IMRaD articles from BMJ 1840–2007 and this difference is statistically significant.

In other terms, within the three scientific corpora, the percentage of certainty progressively increases (from 80% to 91%) and, conversely, the percentage of uncertainty progressively decreases (from 20% to 9%).

Specifically, the results from Study 1 (the comparison between BMJ 1840–2007 no-IMRaD articles and BMJ 1840–2007 IMRaD articles) and Study 3 (the comparison between BMJ 1840–2007 IMRaD articles and BMJ 2013 IMRaD articles) reveal that biomedical scientific writers (1) use UMs significantly less than they did in the past and (2) they place UMs primarily in the Discussion and the Introduction sections (see for example [21; 70–71]).

In other words, the results of our studies suggest that the decreasing uncertainty over time is also related to the different structures of the articles: IMRaD vs. no-IMRaD.

The variables affecting the decreasing of uncertainty are of course multiple, and most of them are often out of experimental control.

Broader explanations could be supposed to be mainly related to the following:

1. Medical progress also linked to the development of new technologies.
2. The different content of the IMRaD articles: commonly, high levels of confidence are assigned to Randomized Control Trials [73] and to meta-analyses [74].
3. Peer review system (used in order to ensure reliability). Being a control system [75], it may favour the publication of those scientific articles in which the uncertainty is limited (Ernest Hart, editor of the BMJ, was one of the first editors to implement a peer-review system). Biomedical articles require an imbalance between certainty and uncertainty in favour of the former. To be published, the submitted scientific biomedical articles need in principle, among other things, to be neither too certain nor too uncertain.
4. Impact Factor (IF): as claimed by Gross & Chesley ([73]: 93) “A higher journal impact factor led to lower amounts of hedging.” From 1990 to 2017, the IF of the BMJ increased from 3.29 to 23.562 (https://www.ncbi.nlm.nih.gov/pmc/articles/PMC1113164/; https://bit.ly/2OY43u6). The decreasing uncertainty in BMJ papers is inversely proportional to the increasing of IF.

### Scientific and popular corpora

Within the same time period (2013), we considered *genre* as the main variable under observation. On the basis of the results from Study 2 (Uncertainty in BMJ 2013) and Study 4 (Uncertainty in Discover 2013), in Study 5, we compared the two corpora. The percentage of certainty and uncertainty in BMJ 2013 is 91% and 9% respectively, while in Discover 2013, it is 88% and 12%. This means that the uncertainty communicated in Discover 2013 is 3 percentage points more than that in BMJ 2013. This difference, consistent with that of [23–24], is statistically significant.

In other terms, the results of our studies confirm the research hypothesis that genre is, among others, a variable affecting the proportion of certainty and uncertainty.

Broader explanations could be supposed to be mainly related to the following:

1. Different writers for different readers: scientific articles are written by specialist writers (scientists and scholars who usually are the authors of the studies presented in the articles) to specialist readers (scientists and scholars), i.e., for the scientific community (peer-to-peer communication). Popular articles are, on the other hand, written by specialist writers (science journalists) for non-specialist readers [27, 29, 72].
2. Different aims: scientific articles aim to share and discuss new findings within the scientific community. Such new findings can determine contrary outcomes in terms of health policies, clinical practice, etc. On the other hand, popular articles aim to spread scientific knowledge within the non-experts community in order to make people aware and responsible in assuming subsequent attitudes and behaviours.
3. Different structures of the articles [76–77]: scientific articles have a more rigorous, fixed, structure (IMRaD) in which the author has to present relevant literature on the topic, experimental design, methodology, quantitative results, etc. Popular articles do not have a fixed structure as scientific articles do, since the main purpose of the science journalists is to summarize, simplify, and compare different studies on a specific topic in order to render them understandable to laypeople. Furthermore, scientific writers often present statistical data, meta-analysis, etc. to support their own assumptions, while science journalists largely use direct or indirect quotations of different researchers without adopting a personal position towards them. In other words, science journalists remain uncertain and neutral about different scientific perspectives, leaving the choice to their readers.

The major limitation of the present paper concerns the size of the two corpora, BMJ 2013 and Discover 2013, since each of them consists of only 12 articles. In the future, we intend to enlarge these two corpora in order to further test our results.
